# Supplementary material for: Epigenetic reprogramming by TET enzymes impacts co-transcriptional R-loops
Source: eLife. 2022 Feb 22;11:e69476. doi: 10.7554/eLife.69476 (PMC8896830; doi:10.7554/eLife.69476)
Supplement: Supplementary file 2. — (A) shRNA sequences. (B) Oligonucleotide sequences. (C) Antibodies used in this study. (D) g-Blocks sequences. (E) S9.6 electrophoretic mobility shift assay (EMSA) oligonucleotides. [file elife-69476-supp2.docx]

Supplementary File 2:

A: shRNA sequence.

| Gene knockdown | shRNA sequence |
| --- | --- |
| *Tet3* | tgctgttgacagtgagcgcgcagtgtgtattcctaccatttagtgaagccacagatgtaaatggtaggaatacacactgcttgcctactgcctcgga |

B: Oligonucleotide sequences.

| Primers | Sequence |
| --- | --- |
| M13 FOR long | GTTTTCCCAGTCACGACGTTGT |
| M13 REV long | AACAGCTATGACCATGATTACGCCA |
| Tet1 Transcript FW | GAAGGTATCCCTCGCCTGAT |
| Tet1 Transcript RV | CCACGAACAGCCAAAGGAGA |
| Tet2 Transcript FW | GTCAACATGCCAGGAGGATTC |
| Tet2 Transcript RV | TTGCGGGTGAGCCTCAGATG |
| Tet3 Transcript FW | ACACCCTCTACCAGGAGCTT |
| Tet3 Transcript RV | GCAGCCGTTGAAGTACATGC |
| Smad9_DRIP_RNA FW | CACAGCGAGTACAACCCTCA |
| Smad9_DRIP_RNA RV | ATGGAGACTGCGGAAACACA |
| Diexf_DRIP_RNA FW | ATGCGATAGCTCTTGGGAGG |
| Diexf_DRIP_RNA RV | TTCAACCCGCCCTTCCATTT |
| Tom1l1_DRIP FW | CACATGGGTCTTACAGACAG |
| Tom1l1_DRIP RV | GAGTTTGGGATGCTGGTGAT |
| Tom1l1_RNA FW | TTCTGTTCTGGGTCTCCAGC |
| Tom1l1_RNA RV | ATGTGCGTGCAGAACTGTGG |
| Slc8a1_DRIP_RNA FW | GTCCATTGCTGCCATCTACCA |
| Slc8a1_DRIP_RNA RV | GAAGATGTGAGGAGCTTGGCA |
| Actb_DRIP_RNA FW | GAACCGCTCGTTGCCAATAG |
| Actb_DRIP_RNA RV | CACCACAGCTGAGAGGGAAA |
| Hprt1_DRIP_RNA FW | GTCATGAAGGAGATGGGAGG |
| Hprt1_DRIP_RNA RV | ATGTAATCCAGCAGGTCAGC |
| Palm2_DRIP FW | CAGCTTTATCCTGGGCGTGA |
| Palm2_DRIP RV | TCATCCCGCATCCTATGCAC |
| Palm2_RNA FW | ACAATGGCCTCCTCGCTGAT |
| Palm2_RNA RV | ATCACAGCAGTTGGCCTCCA |
| Gpr180_DRIP FW | ACCTTGATACACGCGCTCTT |
| Gpr180_DRIP RV | TGCTGACCTTGACAATCGAC |
| Gpr180_RNA FW | AGACGGAGCACAACCTCACA |
| Gpr180_RNA RV | CGGGTTGAGGAGCACCATTT |
| Srpk1_DRIP FW | TGCTCACAACGTCTTACCCA |
| Srpk1_DRIP RV | CCTCCTGAGACCAAGATTCT |
| Srpk1_RNA FW | AGTCATTGGGGTCTTCCTGC |
| Srpk1_RNA RV | TGAAACTCAGCACCGAGGCT |
| Hip1r_DRIP FW | TGCATGACTATCAGCGGTAC |
| Hip1r_DRIP RV | TTATGGAGCTGTCGGCCAAT |
| Hip1r_RNA FW | AGGGAGCCTTTACCTTCTGG |
| Hip1r_RNA RV | GAGGACCTTGTGAAGGACGT |
| B2m_DRIP_RNA FW | ACGTAACACAGTTCCACCCG |
| B2m_DRIP_RNA RV | TCAGTCTCAGTGGGGGTGAA |
| APOE Pair A FW | GCTGCGTTGCTGGTCACATT |
| APOE Pair A RV | CAGGAGGTTGAGGTGAGGAT |
| APOE Pair B_1 FW | GCCCGAGCTGCGCCAG |
| APOE Pair B_1 RV | ACAGTGTCTGCACCCAGC |
| APOE Pair B_2_mRNA FW | GGCAGAGCGGCCAGCG |
| APOE Pair B_2_mRNA RV | CTCCTCCTGCACCTGCTC |
| APOE Pair C_1 FW | GCCTACAAATCGGAACTGGA |
| APOE Pair C_1 RV | CAGCTCCTCGGTGCTCTG |
| APOE Pair C_2 FW | CCGTTCCTTCTCTCCCTCTT |
| APOE Pair C_2 RV | TCCAGTTCCGATTTGTAGGC |
| APOE Pair D FW | TGAAGGAGCAGGTGGCGGA |
| APOE Pair D RV | CTGGCGCTGCATGTCTTCCA |
| Pou5f1 FW | GAAGCCGACAACAATGAGAACC |
| Pou5f1 RV | CTCCAGACTCCACCTCACACG |
| Sox17 FW | ACAACGCAGAGCTAAGCAAGAT |
| Sox17 RV | GTACTTGTAGTTGGGGTGGTCCT |
| Sox6 FW | TCAACCTGCCAAACAAAAGC |
| Sox6 RV | GCTGGATCTGTTCTCGCATC |
| Dll1 FW | GCAGGACCTTCTTTCGCGTAT |
| Dll1 RV | AAGGGGAATCGGATGGGGTT |
| U6 snRNA FW | GCTTCGGCAGCACATATACTA |
| U6 snRNA RV | AAATATGGAACGCTTCACGA |
| Gapdh FW | AACTTTGGCATTGTGGAAGG |
| Gapdh RV | ACACATTGGGGGTAGGAACA |

C: Antibodies used in this study.

| Product | Concentrations | Company/ Cat. No. | Notes |
| --- | --- | --- | --- |
| S9.6 | 5ug/ IP; 1:1000 (DB) | Millipore; MABE1095 | Anti-DNA:RNA hybrid antibody used to detect R-loops |
| dsDNA | 1:1000 (DB) | Santa Cruz; sc-58749 | Anti-dsDNA specific antibody (HYB331-01) |
| 5hmC | 5ug/ IP; 1:1000 (DB) | Active Motif; 39791 | 5-hydroxymethylcytosine antibody |
| 5mC | 5ug/ IP; 1:500 (DB) | Active Motif; 61255 | 5-methylcytosine antibody |

D: g-blocks sequences.

| g-blocks |
| --- |
| *ACTB P1* |
| CTGACAACCGGTGTTTTCCCAGTCACGACGTTGTTAATACGACTCACTATAGGGTTACCCAGAGTGCAGGTGTGTGGAGATCCCTCCTGCCTTGACATTGAGCAGCCTTAGAGGGTGGGGGAGGCTCAGGGGTCAGGTCTCTGTTCCTGCTTATTGGGGAGTTCCTGGCCTGGCCCTTCTATGTCTCCCCAGGTACCCCAGTTTTTCTGGGTTCACCCAGAGTGCAGATGCTTGAGGAGGTGGGAAGGGACTATTTGGGGGTGTCTGGCTCAGGTGCCATGCCTCACTGGGGCTGGTTGGCACCTGCATTTCCTGGGAGTGGGGCTGTCTCAGGGTAGCTGGGCACGGTGTTCCCTTGAGTGGGGGTGTAGTGGGTGTTCCTAGCTGCCACGCCTTTGCCTTCACCTATGGGATCGTGGCTGTCAGCCTTGAGGGTCAGCCTGGCCCAGGCTCCTGGCGTAATCATGGTCATAGCTGTTTGTACACTGACA |
| *ACTB P2* |
| CTGACAACCGGTGTTTTCCCAGTCACGACGTTGTTAATACGACTCACTATAGGGGGGACTATTTGGGGGTGTCTGGCTCAGGTGCCATGCCTCACTGGGGCTGGTTGGCACCTGCATTTCCTGGGAGTGGGGCTGTCTCAGGGTAGCTGGGCACGGTGTTCCCTTGAGTGGGGGTGTAGTGGGTGTTCCTAGCTGCCACGCCTTTGCCTTCACCTATGGGATCGTGGCTGTCAGCCTTGAGGGTCAGCCTGGCCCAGGCTCCCATAGGCTTAGGAGAGGCCGCAATTCCTACCTGTTCATCCAGACAGAGGGGGACCTGGAATCAAAGTCAAGTTGGGGTAGGGGGTCCATGGGGCCATATCTGGCCTGCAGACAGCTCTGGTTAGCTATGGGCTGAGGTCTGGATTCTGCCTTGTGACTGGAGACTGGGCGCCATCCCGTGGCCTCTGAGGGCTGGCGTAATCATGGTCATAGCTGTTTGTACACTGACA |
| *APOE* |
| CTGACAACCGGTGGTTTTCCCAGTCACGACGTTGTAATACGACTCACTATAGGGCCGGTGAGAAGCGCAGTCGGGGGCACGGGGATGAGCTCAGGGGCCTCTAGAAAGAGCTGGGACCCTGGGAACCCCTGGCCTCCAGGTAGTCTCAGGAGAGCTACTCGGGGTCGGGCTTGGGGAGAGGAGGAGCGGGGGTGAGGCAAGCAGCAGGGGACTGGACCTGGGAAGGGCTGGGCAGCAGAGACGACCCGACCCGCTAGAAGGTGGGGTGGGGAGAGCAGCTGGACTGGGATGTAAGCCATAGCAGGACTCCACGAGTTGTCACTATCATTTATCGAGCACCTACTGGGTGTCCCCAGTGTCCTCAGATCTCCATAACTGGGGAGCCAGGGGCAGCGACACGGTAGCTAGCCGTCGATTGGAGAACTTTAAAATGAGGACTGAATTAGCTCATAAATGGCGTAATCATGGTCATAGCTGTTTGTACACTGACA |

E: S9.6 EMSA oligonucleotides.

| Single stranded oligonucleotide | Sequence |
| --- | --- |
| ssDNA_S9.6 EMSA | GCTGTCAGAC |
| ssRNA_S9.6 EMSA | GUCUGACAGC |
