# Supplementary material for: Epigenetic reprogramming by TET enzymes impacts co-transcriptional R-loops
Source: eLife. 2022 Feb 22;11:e69476. doi: 10.7554/eLife.69476 (PMC8896830; doi:10.7554/eLife.69476)
Supplement: Source data 1. [file elife-69476-data1.pptx]

## Slide 1
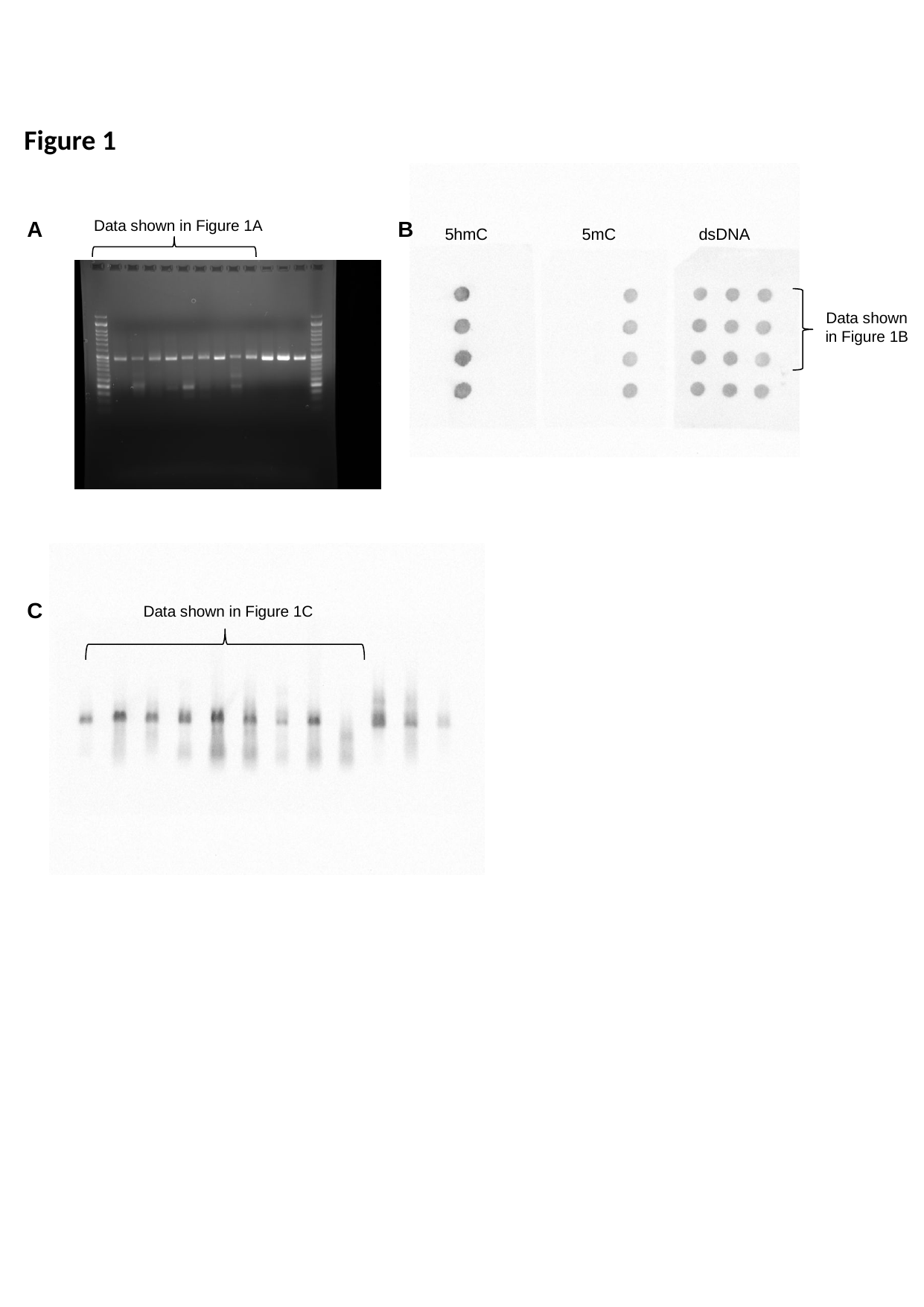

Figure 1
A
Data shown in Figure 1A
B
5hmC
dsDNA
5mC
Data shown in Figure 1B
C
Data shown in Figure 1C

## Slide 2
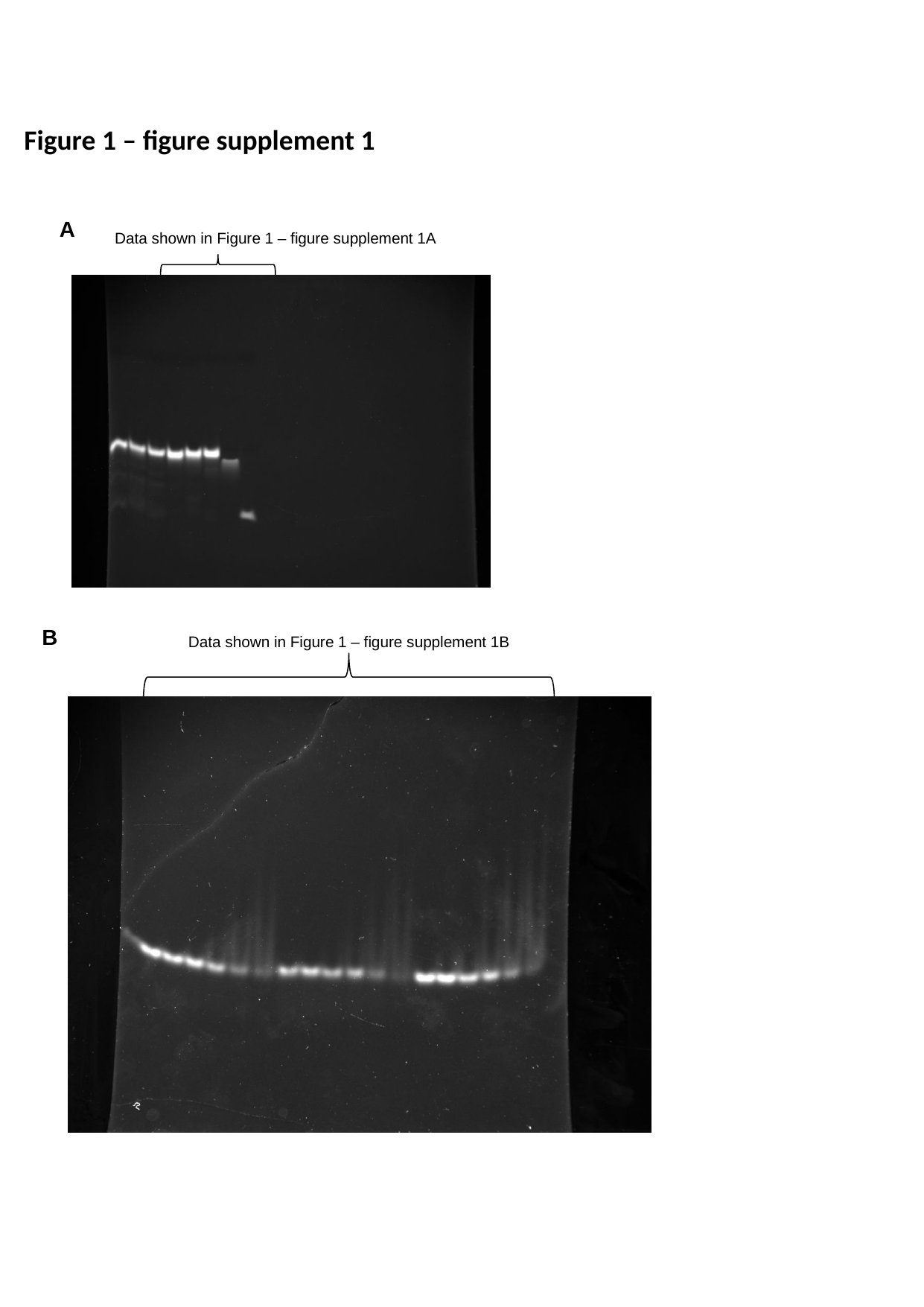

Figure 1 – figure supplement 1
A
Data shown in Figure 1 – figure supplement 1A
B
Data shown in Figure 1 – figure supplement 1B
