# Supplementary material for: Epigenetic reprogramming by TET enzymes impacts co-transcriptional R-loops
Source: eLife. 2022 Feb 22;11:e69476. doi: 10.7554/eLife.69476 (PMC8896830; doi:10.7554/eLife.69476)
Supplement: Source data 2. [file elife-69476-data2.pptx]

## Slide 1
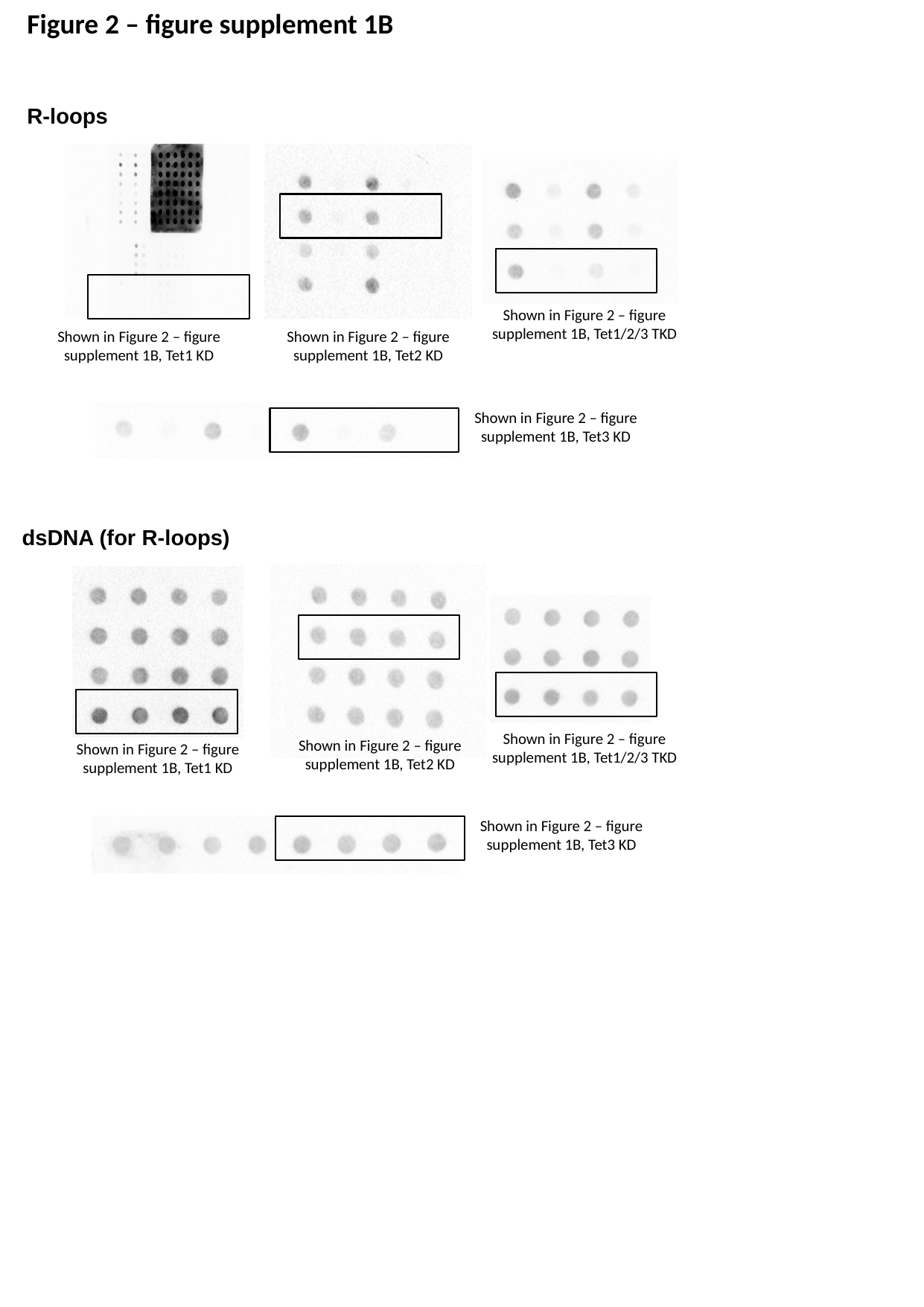

Figure 2 – figure supplement 1B
R-loops
Shown in Figure 2 – figure supplement 1B, Tet1/2/3 TKD
Shown in Figure 2 – figure supplement 1B, Tet1 KD
Shown in Figure 2 – figure supplement 1B, Tet2 KD
Shown in Figure 2 – figure supplement 1B, Tet3 KD
dsDNA (for R-loops)
Shown in Figure 2 – figure supplement 1B, Tet1/2/3 TKD
Shown in Figure 2 – figure supplement 1B, Tet2 KD
Shown in Figure 2 – figure supplement 1B, Tet1 KD
Shown in Figure 2 – figure supplement 1B, Tet3 KD

## Slide 2
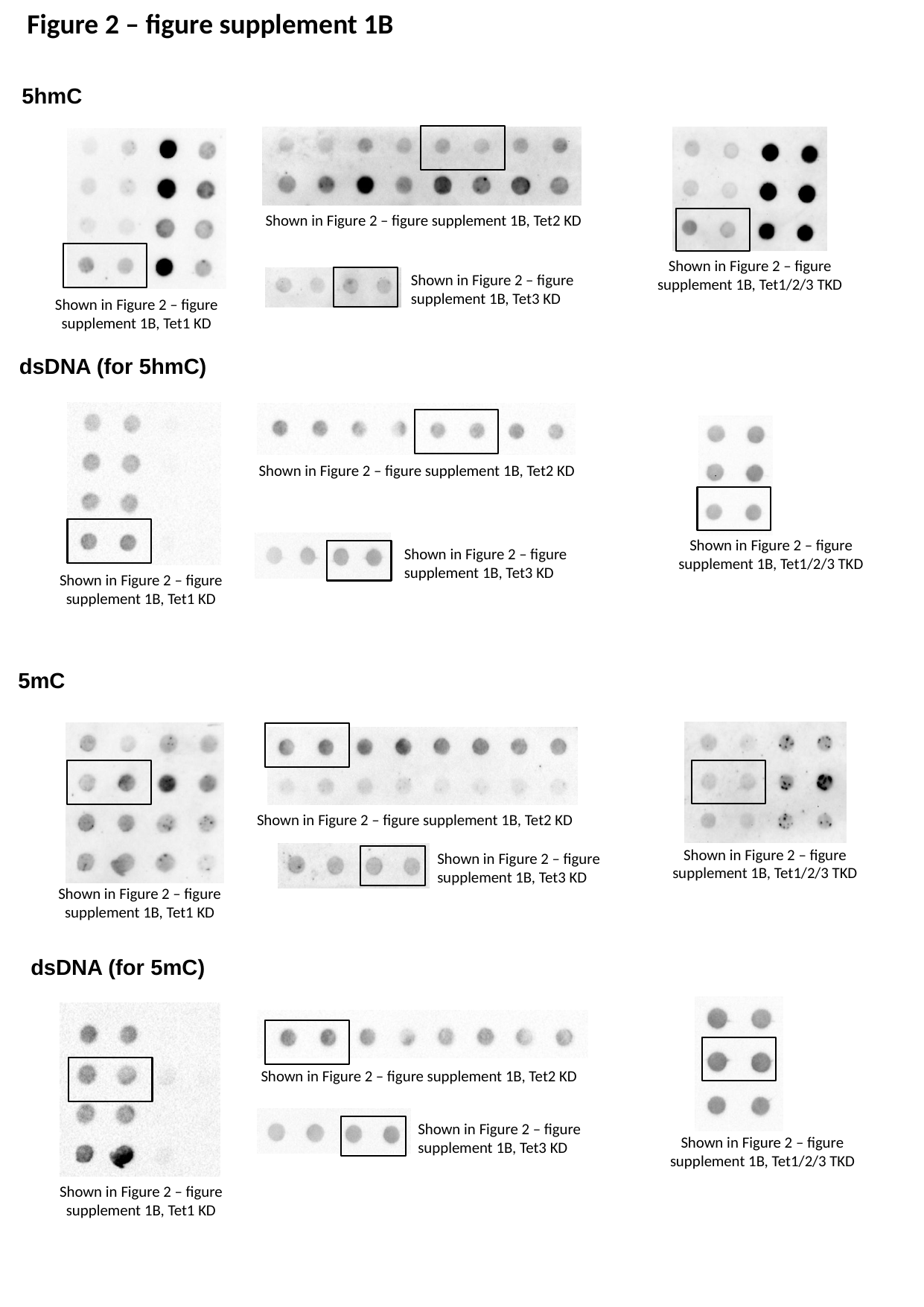

Figure 2 – figure supplement 1B
5hmC
Shown in Figure 2 – figure supplement 1B, Tet2 KD
Shown in Figure 2 – figure supplement 1B, Tet1/2/3 TKD
Shown in Figure 2 – figure supplement 1B, Tet3 KD
Shown in Figure 2 – figure supplement 1B, Tet1 KD
dsDNA (for 5hmC)
Shown in Figure 2 – figure supplement 1B, Tet2 KD
Shown in Figure 2 – figure supplement 1B, Tet1/2/3 TKD
Shown in Figure 2 – figure supplement 1B, Tet3 KD
Shown in Figure 2 – figure supplement 1B, Tet1 KD
5mC
Shown in Figure 2 – figure supplement 1B, Tet2 KD
Shown in Figure 2 – figure supplement 1B, Tet1/2/3 TKD
Shown in Figure 2 – figure supplement 1B, Tet3 KD
Shown in Figure 2 – figure supplement 1B, Tet1 KD
dsDNA (for 5mC)
Shown in Figure 2 – figure supplement 1B, Tet2 KD
Shown in Figure 2 – figure supplement 1B, Tet3 KD
Shown in Figure 2 – figure supplement 1B, Tet1/2/3 TKD
Shown in Figure 2 – figure supplement 1B, Tet1 KD

## Slide 3
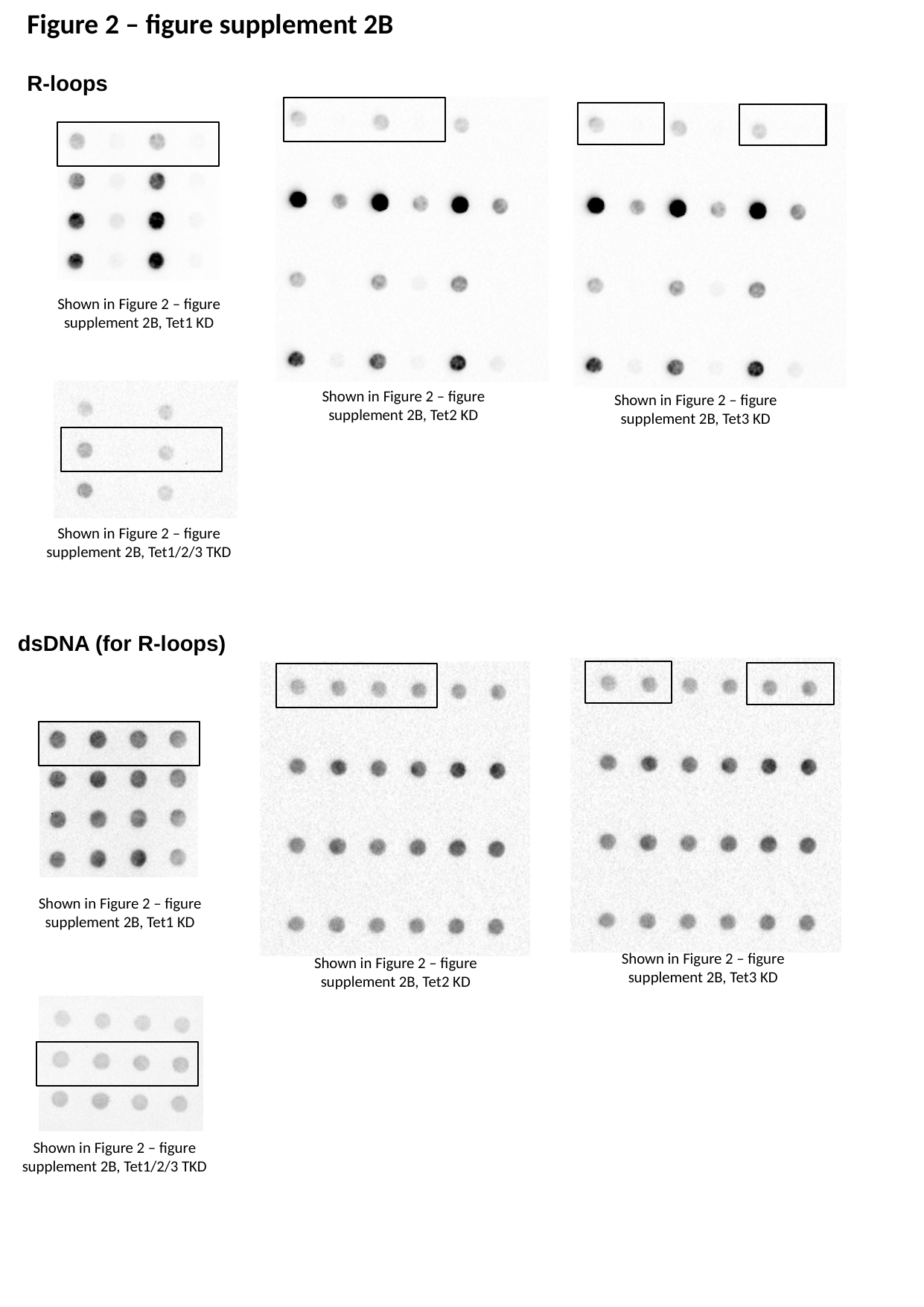

Figure 2 – figure supplement 2B
R-loops
Shown in Figure 2 – figure supplement 2B, Tet1 KD
Shown in Figure 2 – figure supplement 2B, Tet2 KD
Shown in Figure 2 – figure supplement 2B, Tet3 KD
Shown in Figure 2 – figure supplement 2B, Tet1/2/3 TKD
dsDNA (for R-loops)
Shown in Figure 2 – figure supplement 2B, Tet1 KD
Shown in Figure 2 – figure supplement 2B, Tet3 KD
Shown in Figure 2 – figure supplement 2B, Tet2 KD
Shown in Figure 2 – figure supplement 2B, Tet1/2/3 TKD

## Slide 4
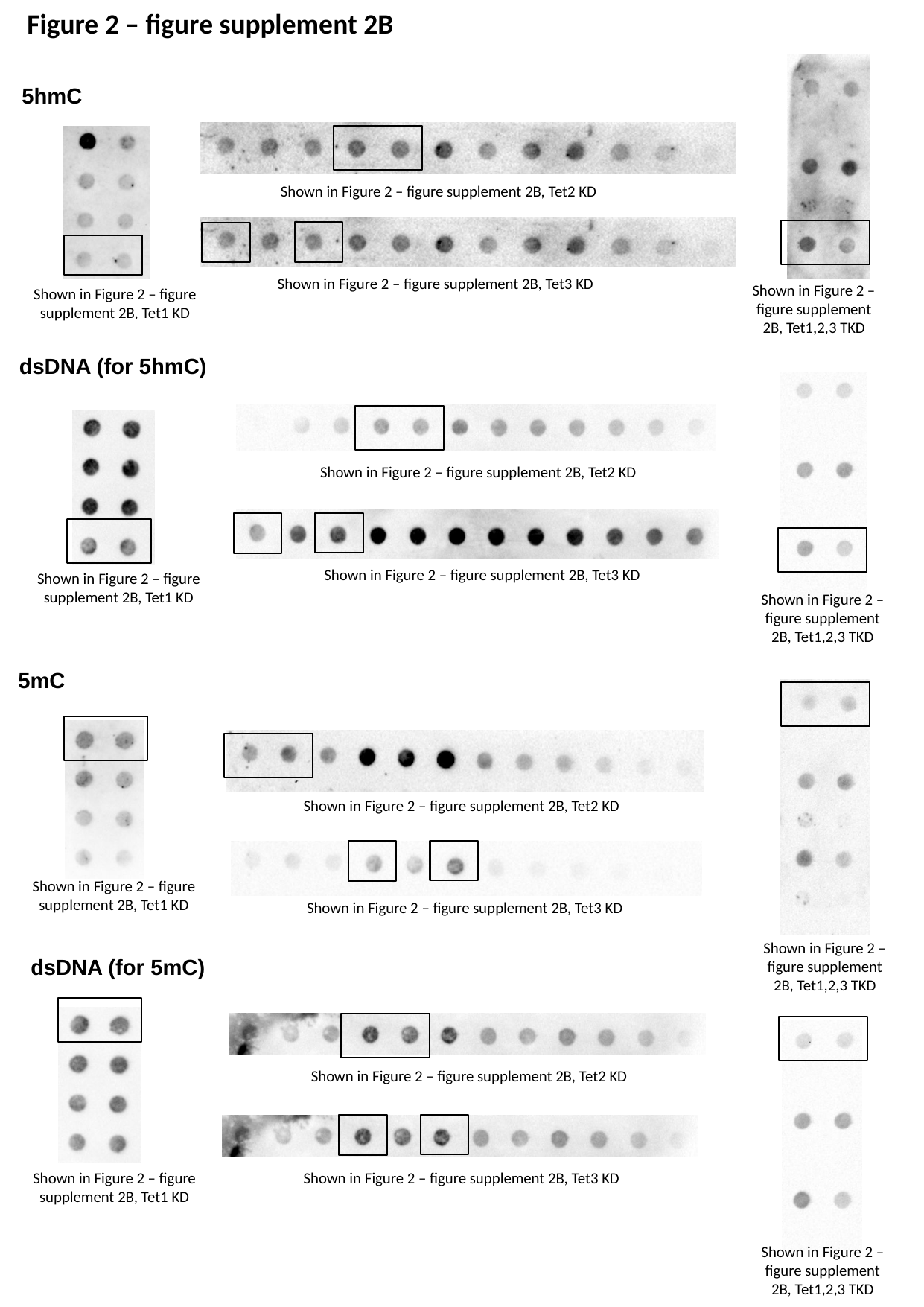

Figure 2 – figure supplement 2B
5hmC
Shown in Figure 2 – figure supplement 2B, Tet2 KD
Shown in Figure 2 – figure supplement 2B, Tet3 KD
Shown in Figure 2 – figure supplement 2B, Tet1,2,3 TKD
Shown in Figure 2 – figure supplement 2B, Tet1 KD
dsDNA (for 5hmC)
Shown in Figure 2 – figure supplement 2B, Tet2 KD
Shown in Figure 2 – figure supplement 2B, Tet3 KD
Shown in Figure 2 – figure supplement 2B, Tet1 KD
Shown in Figure 2 – figure supplement 2B, Tet1,2,3 TKD
5mC
Shown in Figure 2 – figure supplement 2B, Tet2 KD
Shown in Figure 2 – figure supplement 2B, Tet1 KD
Shown in Figure 2 – figure supplement 2B, Tet3 KD
Shown in Figure 2 – figure supplement 2B, Tet1,2,3 TKD
dsDNA (for 5mC)
Shown in Figure 2 – figure supplement 2B, Tet2 KD
Shown in Figure 2 – figure supplement 2B, Tet1 KD
Shown in Figure 2 – figure supplement 2B, Tet3 KD
Shown in Figure 2 – figure supplement 2B, Tet1,2,3 TKD
